# Revamping Password Security: Leveraging Mnemonics for Enhanced Authentication

**DOI:** 10.1007/s10207-026-01278-2

**Published:** 2026-07-15

**Authors:** Hannah Ammah Serwaah Adjei, Davide Giovanetti, Miguel Vargas Martin, Matteo Dell’Amico, Paolo Di Prodi

**Affiliations:** 1 Ontario Tech University, Oshawa, Canada; 2 University of Genoa, Genoa, Italy; 3 CrowdStrike Incorporated, London, United Kingdom

**Keywords:** Password, Honeyword, Password manager, Security, Usability, Authentication

## Abstract

Authentication systems often rely on individuals’ ability to remember and accurately input complex passwords in digital environments. However, this task becomes impractical as the demand for higher security measures increases. Researchers have proposed several methods to improve password authentication over the years, yet most methods come with a trade-off between security and usability. Password managers are a well-known solution to make password security usable, but they introduce a widely discussed risk: if a password-manager (PM) vault is compromised, many accounts can be affected at once. This work augments passwords stored in PMs with a user-held secret to improve resilience in a vault-only compromise setting. The user’s login secret is obtained by combining what is stored in the PM with a mnemonic remembered by the user, while the password stored in the PM serves as a decoy that can trigger an alarm indicating potential PM compromise. We conducted a user study using Mindlock, a browser-extension prototype that acts as a mnemonic application interface, to evaluate feasibility and usability in real login routines. Our study provides preliminary evidence that users can adopt mnemonic-based augmentation with limited impact on usability, and we discuss the security implications under a rate-limited online threat model.

## Introduction

Passwords remain the most widely used first line of defense against unauthorized access to personal and professional data. Despite advances in cybersecurity, password-based authentication remains ubiquitous, securing everything from websites to financial services. Their popularity stems from low technical requirements, yet reliance on passwords introduces notable security and usability challenges.

Password complexity poses a major dilemma. Guidelines recommend mixing letters, numbers, and special characters, but such complexity makes passwords difficult to remember, especially when managing multiple accounts. This often leads to password reuse, a common security risk that can cause widespread breaches if one account is compromised. Users tend to choose simple, predictable passwords to ease memorization, increasing vulnerability to attacks. Prior studies [1, 2] recommend emphasizing password length over complexity to improve security without sacrificing memorability.

As complexity and randomness increase, usability suffers. Password fatigue, caused by managing many complex credentials, often leads to poor practices and lockouts. Strong yet memorable passwords remain a major challenge for real-world authentication.

To address these issues, strategies such as meaningful passwords [3], concatenating random words [4], pronounceable passwords [5, 6], helper cards [7], associative memory aids [8], graphical passwords [9], hint images [10, 11], and user-defined rules [12–14] have been proposed. Many of these methods remain vulnerable to attacks or depend heavily on user behavior. For instance, while Jeyaraman et al. [15] proposed textual hints to aid memorization, their approach is limited to alphabetical passwords and needs human intervention to handle digits and special characters.

In parallel, password managers (PMs) have become a practical response to password fatigue by storing and autofilling high-entropy, site-specific passwords. Yet they also introduce a well-known concern: if an attacker gains access to a user’s vault (e.g., by obtaining or guessing the master password, or via a cloud-backed vault exposure), many accounts can be affected at once. For example, in the 2022 LastPass security incident, customer vault backups were reported as accessed and exfiltrated, showing that a vault can be exposed without an attacker first taking control of a user’s device [16, 17]. Similar risks have appeared in other password-manager ecosystems as well [18]. This paper focuses on this vault-only compromise setting: the attacker obtains the PM contents but does not observe the user’s device during login.

This research proposes a mnemonic-based augmentation that integrates with password managers and aims to improve security with a limited impact on usability. When registering for a service, users can opt in to the security-enhanced system, with the default remaining traditional password-based authentication. Users who opt in store a *decoy password* in their PM, while their actual login secret is obtained by modifying the base password through a *mnemonic trick* (e.g., appending or prepending a short secret to the password stored in the PM).

Users adopting our system log in by modifying the password field that is autocompleted by the PM according to their mnemonic trick. If the decoy password is used, this may be a sign of PM compromise, and the user is alerted (e.g., via an automated phone call). Under the vault-only model, an attacker who steals the vault learns only the decoy password and must still guess the mnemonic (and the trick) through an online interface that is typically rate-limited; in practice, online guessing is throttled, so even modest mnemonic secrecy can meaningfully raise the cost of attack in this setting [19, 20] compared to submitting the PM-stored password directly. At minimum, our security posture is similar to that of using PM-generated passwords without our countermeasure, and under vault-only compromise it is strictly better because the PM does not reveal the actual login secret. We do not claim protection under full endpoint compromise (e.g., keyloggers, malicious extensions, or DOM injection).

Since the system is opt-in, an attacker that compromises the PM but not the service itself has no way of knowing that the passwords in the PM are decoys. This raises the probability that an unaware attacker triggers the warning.

Our proposal bears some resemblance to honeywords, another approach using decoy passwords whose usage will trigger a breach alert [21]. Unlike our approach, honeywords require changing the format of password databases, hence making attackers who breach into it aware that the countermeasure is in place.

Our study evaluates the usability and memorability of mnemonic-based augmentation in real password-manager workflows, and discusses feasibility under the scoped threat model in Section 4. The following are the main research questions that we aim to address.

1. **RQ1:** What is the trade-off between security and usability introduced by the proposed methods (under the scoped threat model)?
2. **RQ2:** Are the proposed mnemonic techniques a practical way to simplify password use in password-manager workflows?
3. **RQ3:** Under a vault-only, rate-limited setting, how does mnemonic augmentation affect the feasibility of raising the bar against unauthorized login attempts, and what risks arise from mnemonic predictability?

To answer these research questions, we conducted a user study with 22 participants to evaluate usability, adoption, and mnemonic-use patterns. In summary, the main contributions and findings include:

- **Mnemonic-based augmentation for vault-only compromise:** We propose a mnemonic-based method that augments a password-manager-stored base password and treats the base password as a decoy in the intended deployable design to support compromise detection.
- **Prototype and usability evidence:** We design Mindlock, a browser extension prototype used to evaluate usability and adoption of mnemonic tricks in real login routines (with unmodified websites).
- **Memorability and user behavior insights:** Our results provide evidence that participants can adopt simple mnemonic tricks over time, and we characterize preferences (e.g., prefix vs. suffix) and common failure modes.
- **Risks and mitigations:** We report that mnemonics alone are often predictable (zxcvbn), and we discuss lightweight mitigations (blacklists, strength feedback, minimum policy, and guidance on reuse) needed for deployment.

## Related Work

This section reviews literature relevant to our study, focusing on mnemonic techniques and honeywords for improving password security and usability. We also position our contribution relative to password-manager practices and recent honeyword improvements.

### Overview of Password Security

Password security remains a key topic in cybersecurity research, reflecting ongoing concerns about the weaknesses in user-managed authentication methods. Despite the availability of robust password management tools, users often exhibit insecure habits. A common example is password reuse [22], which can lead to widespread breaches if one account is compromised [23]. Social engineering [24, 25] also poses a serious threat by exploiting human behavior rather than technical flaws. This highlights the need for continuous education [26] and awareness of social engineering tactics [27].

Password managers are often recommended as a practical response to password fatigue, since they can store and autofill high-entropy, site-specific passwords. At the same time, a password-manager vault compromise can have broad impact because it exposes many stored secrets at once. Our work focuses on this *vault-only compromise* setting and proposes a lightweight augmentation that adds a user-held component without requiring users to abandon the password-manager workflow.

### Mnemonic Techniques

Mnemonic techniques are strategies designed to help individuals recall information accurately. Numerous studies have explored these techniques. One popular method is the “method of loci” [8], where learners mentally place information along a familiar path. While effective for memorizing lists, Sousa et al. [28] note that complexity and cognitive demands may reduce its effectiveness without strict adherence.

Radovic et al. [29] highlight the role of mnemonic acronyms in retaining procedural steps, Bellezza et al. [30] point out that no single technique is universally effective, emphasizing the need for careful application based on context. Other techniques include rhymes and songs [31], and visual imagery [32], which help make abstract content more memorable.

Mnemonics also appear in password creation. The xkcd-inspired “correct horse battery staple” [33, 34] illustrates how combining words can yield passwords that are easier to remember than fully random strings. Our work differs in that we do not aim to replace password managers; instead, we use a short user-held mnemonic component as an augmentation in a setting where the base password is already stored by the password manager.

### Honeywords and recent improvements

Honeywords [21] store decoy passwords alongside real passwords so that a *honeychecker* can flag login attempts using decoys. While effective in principle, honeyword schemes can suffer from *distinguishability* (some decoys look less plausible than the real password) [35] and from attacker strategies that exploit structure or selection bias [36]. Recent work has proposed improvements such as typo-tolerant or typo-based honeywords to better model realistic user behavior [37], and language-model-based honeyword generation to produce more plausible decoys [38, 39]. Other recent work explores alternative generation and selection strategies to improve indistinguishability and security guarantees [40].

Positioning of our contribution. Our proposal bears resemblance to honeywords in that it uses a decoy to trigger an alert, but it differs in where detection is anchored. Traditional honeywords require changes to the service’s password database format (e.g., storing multiple candidates per account), which can make the presence of the countermeasure visible to an attacker who compromises the password database. In contrast, we relocate decoy exposure to the password-manager compromise setting: the password manager stores only the base password $B_{p_i}$ (treated as a decoy), while the actual login secret is the enhanced password $E_{p_i}$ that includes a user-held mnemonic. Under vault-only compromise, an attacker who obtains the vault learns $B_{p_i}$ but not $E_{p_i}$, and submitting $B_{p_i}$ in the deployable system can trigger an alarm. This design aims to raise the bar in the vault-only, rate-limited setting while keeping the password-manager workflow largely unchanged.

Building on these insights, the next section presents our system design that combines mnemonic augmentation with decoy-triggered detection.

## System Overview

This section introduces Mindlock, the mnemonic interface that will aid users in creating and appending their mnemonics to the base password. Mindlock is not a part of our proposed system; it is an extension substituted as the mnemonic component of our proposed system, and its primary goal is to evaluate how feasible our method is through a user study. In other words, the purpose of Mindlock is to assess whether our strategy is usable.

### Architecture

The Mindlock extension consists of three main components: **Popup**, **Content Script**, and **Service Worker**. Each serves a distinct role and works together to support the extension’s functionality.

- **Popup:** The popup acts as the user interface, accessible via the browser toolbar. It provides a simple interface for interacting with the extension’s features and settings.
- **Content Script:** This script runs in the context of web pages and interacts with the DOM to detect forms, clicks, and password submissions. It identifies login attempts and sends the entered password to the service worker for mnemonic verification. Based on the response, it either proceeds or interrupts the login.
- **Service Worker:** Running in the background, the service worker handles password verification and syncs data between the popup and content script. It uses browser APIs to ensure secure communication and coordination across components.

### Permissions and Logged Events

Permissions serve as the gateway for Chrome extensions to access essential functionalities and data within the browser environment. By granting permissions, users authorize extensions to perform specific actions or access certain resources, enabling them to enhance browsing experiences and provide valuable features. Permissions in Chrome extensions are designed to balance functionality with security and privacy considerations.

- **ActiveTab:** Provides the extension with temporary access to information about the currently active tab.
- **Scripting:** Enables the injection of code into the current page and the return of a value to the popup scope. This facilitates adjustments to the layout of the pop-up based on the presence of a form.
- **Storage:** Utilized for storing mnemonic tricks and logs.
- **Downloads:** Grants permission for file downloads necessary for obtaining the final report.

In addition to the above permissions, Mindlock records the following *logged events* locally during the study:

- **Mnemonic creation:** Captures the mnemonic technique and its associated component, the creation timestamp, and, if the mnemonic is domain-specific, the hashed domain related to it.
- **Site enabling/disabling:** Records the hashed domain in question, the timestamp of the user’s action, and an indicator showing whether the domain was enabled or disabled.
- **Login attempts:** Tracks login attempts, including the timestamp, the hashed domain, and the outcome of the attempt (successful or unsuccessful).

### Mnemonic Integration Process

**Fig. 1:**
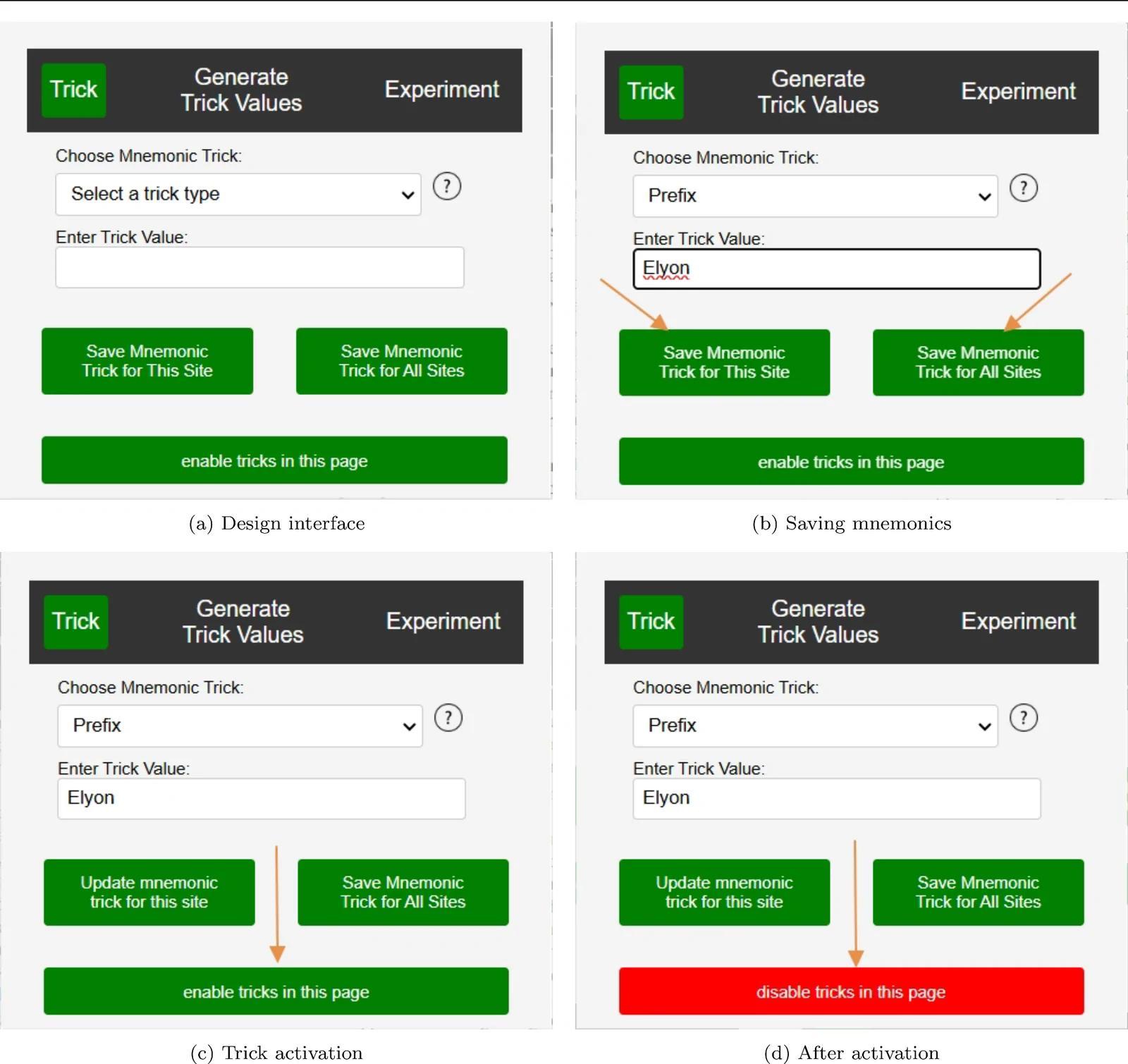
Mindlock interface steps

Upon installing the Mindlock extension from the Google Chrome store and accessing its features, users are greeted with an intuitive interface designed to smoothen the integration of mnemonics into their existing passwords. Figure 1 shows the interface design of Mindlock.

1. **Selecting the Mnemonic Trick Type:** Users can choose from three types of mnemonic tricks.
  **Prefix trick:** Append a mnemonic to the beginning of an existing password. For example, mnemonic “Start” and password “Memshach” yields “StartMemshach”.
  **Suffix trick:** Append a mnemonic at the end of the password. Using the same example, the enhanced-looking input would be “MemshachStart”.
  **Repetition trick:** Users choose a text and a repetition number *n* to append to the beginning of their password and after every *n* characters. For example, choosing the text “Hello” with $n{=}3$ and applying it to “Simple” results in “HelloSimHellopleHello”.
2. **Entering and Saving the Mnemonic Trick:**
  Users input their chosen mnemonic into the “Enter Text” field.
  They can save it for the current site or across all sites, providing flexibility in how they apply mnemonics.
3. **Applying the Mnemonic Trick:**
  Users enable the mnemonic on the current page by clicking the “enable tricks in this page” button.

To ensure mnemonic validity, users must provide components with a minimum of five characters for prefixes, suffixes, or repetitions. Real-time feedback is offered to guide users when inputs do not meet these constraints. When selecting a technique, users can click a question mark for a brief explanation, ensuring they understand the method before applying it. Overall, this process supports users in creating and consistently applying mnemonic tricks, allowing us to evaluate usability in real login routines.

### Prototype vs. Deployable System

Our paper describes two closely related systems that serve different purposes: (i) an intended deployable architecture where the service verifies the enhanced password, and (ii) the Mindlock study prototype used only to evaluate usability without changing any website.

Deployable system. In the intended deployment, the user’s login secret is the enhanced password $E_{p_i}$, formed by applying a mnemonic trick to the base password $B_{p_i}$ using a user-held mnemonic $M_i$. The server verifies $E_{p_i}$ and triggers an alarm if $B_{p_i}$ is submitted (treating $B_{p_i}$ as a decoy).

Study prototype. In our user study, websites were unmodified and continued to verify the original password $B_{p_i}$. Mindlock therefore intercepts the user’s enhanced input, checks that the mnemonic pattern is followed, strips the mnemonic locally, restores $B_{p_i}$, and submits only $B_{p_i}$ to the website. Thus, the study evaluates the usability of mnemonic creation and consistent application, not server-side decoy detection.

Deployable login flow (server verifies $E_{p_i}$*).*

- The password manager autofills the stored base password $B_{p_i}$ (decoy) for the site.
- The user edits the field by applying their mnemonic trick using mnemonic $M_i$ to form $E_{p_i}$ (e.g., prefix/suffix).
- The user submits $E_{p_i}$ to the server.
- The server accepts if the submitted value matches the verifier for $E_{p_i}$; if it matches $B_{p_i}$, the server triggers an alarm.

Study login flow (Mindlock strips mnemonic; website verifies $B_{p_i}$*).*

- The password manager autofills the stored base password $B_{p_i}$ for the site.
- The user edits the field by applying their mnemonic trick (enhanced-looking input).
- Mindlock intercepts the submission event, checks that the expected mnemonic pattern is present, and removes the mnemonic locally.
- Mindlock restores the field to the base password $B_{p_i}$ and submits the form; the website verifies $B_{p_i}$ as usual.

Multi-device implications. In the deployable system, Mindlock is optional: users can apply the mnemonic manually on any device and submit $E_{p_i}$ directly. In the study, Mindlock is required because the website still expects $B_{p_i}$; if the user logs in from a browser/device without the extension during the study, the mnemonic must not be appended to the base password for that site (or the login will fail because the website does not recognize $E_{p_i}$).

### Authentication Mechanism

**Study prototype behavior.** During login, users enter an enhanced-looking password (base password + mnemonic). Mindlock intercepts this input, separates the mnemonic, and passes only the original password to the website. It captures the form’s JavaScript event, restores the base password, and completes the submission. This stripping is necessary because Mindlock is used solely for user study purposes, not as an actual authentication product. Users can activate the extension across multiple domains and assign unique mnemonics.

### Security Features and Requirements

Mindlock checks whether the mnemonic pattern is followed, removes it, and proceeds with the login. Logs are anonymized and contain no personal data; users were advised against using real passwords as mnemonics. All logs are encrypted using AES-256 [41, 42], and domain names are hashed using cyrb53 with a key derived from the user’s Mindlock username and first login timestamp, making navigation history computationally infeasible to reverse-engineer. Mindlock requires at least Google Chrome version 90.0.4606.85 or Chromium version 126.0.6444.0.

While the intended deployable design aims to raise the bar under vault-only compromise, it does not claim protection under endpoint compromise (e.g., keyloggers, malicious extensions, DOM injection, clipboard monitoring) or phishing that captures what a user submits. In the study prototype, Mindlock evaluates usability and consistency of mnemonic application, not end-to-end server-side detection.

**Fig. 2:**
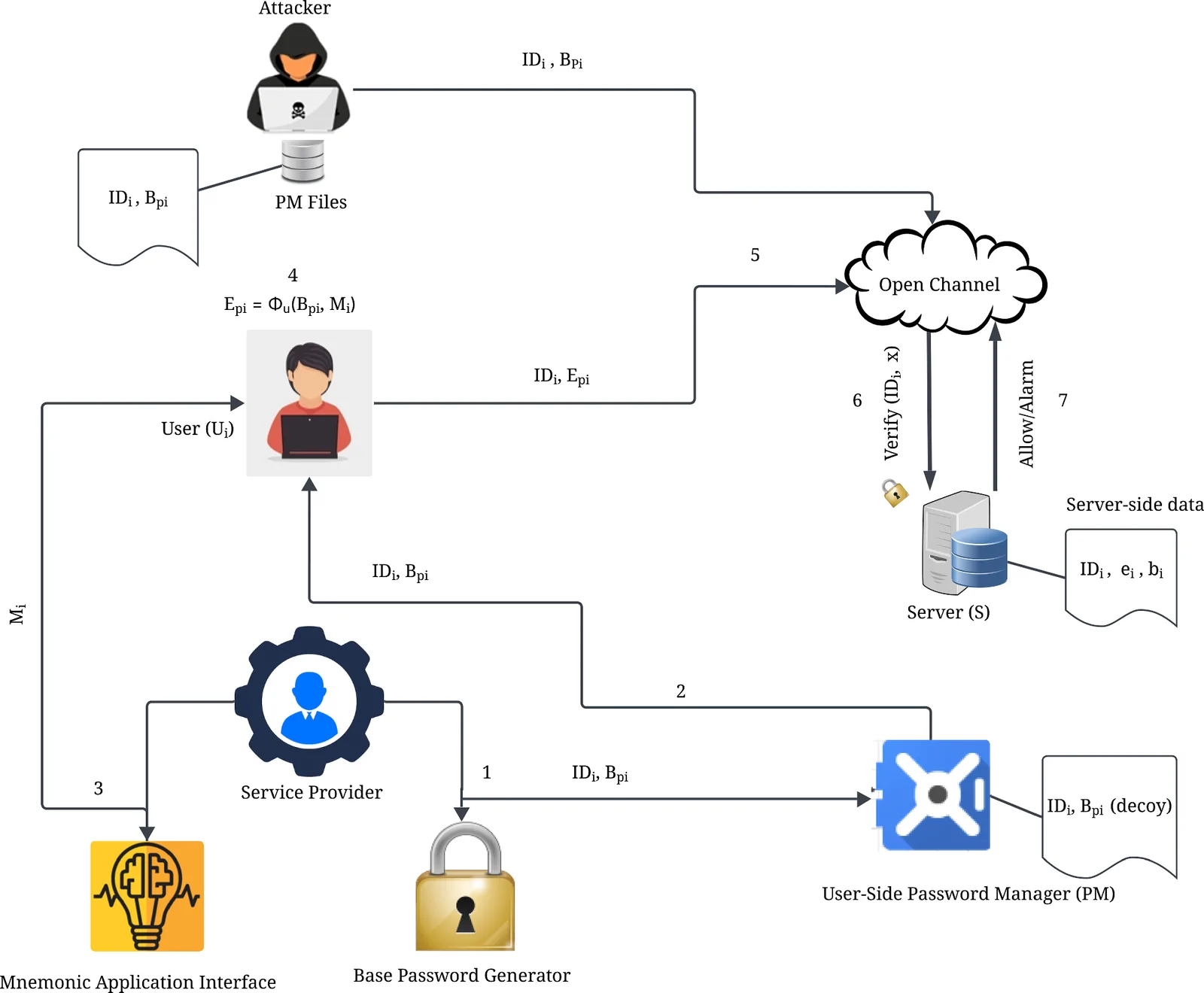
System workflow. (1) Service provider creates a base password stored in the password manager; (2) user retrieves it; (3) user creates a mnemonic; (4) base password and mnemonic are combined; (5) enhanced password is sent to the server; (6) server verifies credentials; (7) an alarm is triggered if a decoy word is detected

## Threat Model and Approach

We adopt Kerckhoffs’s principle: attackers are assumed to know our mechanism and, in the worst case, to know that a user has enabled it. Deployment is *opt-in* (only some users enable the mnemonic layer due to its usability cost), and our security claims are stated for attackers who are aware of enablement. If attackers do not know whether the user has enabled the mechanism, they may be more likely to attempt to log in with the decoy and raise an alarm. Even if they know the system is enabled, our claims still hold under the threat model defined below. Attacker knowledge of adoption (two cases). We consider two cases. (1) The attacker knows the user adopted the mnemonic layer; this is the worst-case framing used in our main analysis. (2) The attacker does not know whether the user adopted the mnemonic layer. In this case, the vault contents do not reveal adoption, so the attacker cannot distinguish accounts protected by our scheme from standard password-manager accounts. For users who did adopt the scheme, attempting to use the vault-stored base password is likely to trigger an alarm. More broadly, this uncertainty can make attackers less willing to attempt online logins to avoid triggering alerts, which can provide residual benefit even for users who did not adopt the scheme.

Secrecy resides only in the user’s mnemonic and its user-specific application. The Service Provider (SP) generates a random base password $B_{p_i}$ via its Password Generator (PG). Using a Mnemonic Application Interface (MAI), the user chooses a mnemonic $M_i$ and the login secret becomes the enhanced password $E_{p_i}$ (Fig. 2). After enrolment, $B_{p_i}$ is intentionally repurposed as a decoy stored in the Password Manager (PM). The server *S* (operated by the SP) stores (i) a standard verifier for $E_{p_i}$ and (ii) a *protected* representation of the account’s decoy used only for detection; the PM never stores $E_{p_i}$.

### Threat model scope and security argument

Our primary target is a vault-only compromise of the password manager (PM): the attacker obtains PM contents (including $B_{p_i}$) but does not observe the user or endpoint during login and is constrained by normal online throttling (rate limits, lockouts, and monitoring). Out of scope are endpoint-compromise threats that directly observe secrets at entry time, including keyloggers, screen capture, malicious browser extensions, DOM-injection malware, and clipboard monitoring. We do not claim protection under full device compromise; as with most PM-based schemes, if the endpoint is fully controlled, the adversary may recover the mnemonic $M_i$.

*Attacker’s task under vault-only compromise.* Under vault-only compromise, the attacker learns the base/decoy password $B_{p_i}$ from the PM. To authenticate without triggering the decoy alarm, the attacker must submit the enhanced password $E_{p_i}$, which requires guessing (i) the mnemonic trick *t* and (ii) the mnemonic $M_i$.

Let *T* denote the set of supported tricks (e.g., prefix and suffix), and let t∈T denote the specific trick chosen for account *i*. Let $G(M_i)$ denote the guessability of the mnemonic under an attacker’s online guessing strategy (capturing the fact that users may choose predictable mnemonics). A simple way to express the attacker’s search space is:

1 $$\begin{aligned} \text {Search space} \;\approx \; |T|\times G(M_i). \end{aligned}$$

This captures the intended security gain: compromising the PM reveals $B_{p_i}$ but does not reveal the actual login secret $E_{p_i}$, so the attacker must additionally guess $M_i$ (and *t*) online.

Implications of mnemonic guessability. Our study measured mnemonic-only strength using zxcvbn and found that many user-chosen mnemonics are predictable. This means $G(M_i)$ may be small for some users, reducing the benefit. For this reason, our security claims are scoped to the vault-only, rate-limited setting, and we recommend mitigations (e.g., mnemonic blacklists, a strength meter, and minimum policy requirements) to *reduce* mnemonic guessability and discourage reuse.

Notation and transform. Let $\mathcal {B}$ be the space of base passwords, $\mathcal {M}$ the space of mnemonics, and $\mathcal {E}$ the space of enhanced passwords. For each user *u*, define the private transform

2 $$\begin{aligned} \phi _u:\ \mathcal {B}\times \mathcal {M}\rightarrow \mathcal {E}. \end{aligned}$$

Given $B_{p_i}\in \mathcal {B}$ and $M_i\in \mathcal {M}$, the login secret is

3 $$\begin{aligned} E_{p_i}=\phi _u(B_{p_i},\,M_i). \end{aligned}$$

The PM only stores the base (decoy) password $B_{p_i}$, using the standard encryption techniques used for traditional passwords.

### Transform and parse rules

We instantiate $\phi _u$ using simple, unambiguous tricks that admit a deterministic parse. We use ‖ to denote string concatenation. Let *T* be the set of supported tricks and let t∈T denote the specific trick chosen for an account. In what follows, $\textsf{parse}_t(\cdot )$ is the client-side procedure that removes the mnemonic and recovers the base password.

Prefix trick. For $t=\textsf{prefix}$, the enhanced password is

4 $$\begin{aligned} E_{p_i} = M_i \,\Vert \, B_{p_i}. \end{aligned}$$

The corresponding parse procedure checks whether the input begins with $M_i$ and, if so, removes that prefix:

$$ \textsf{parse}_{\textsf{prefix}}(E_{p_i}) = B_{p_i}. $$

Suffix trick. For $t=\textsf{suffix}$, the enhanced password is

5 $$\begin{aligned} E_{p_i} = B_{p_i} \,\Vert \, M_i. \end{aligned}$$

The parse procedure checks whether the input ends with $M_i$ and, if so, removes that suffix:

$$ \textsf{parse}_{\textsf{suffix}}(E_{p_i}) = B_{p_i}. $$

Ambiguity and validation. In the study prototype, Mindlock validates that the expected mnemonic $M_i$ appears in the correct position (prefix or suffix) for the selected trick; otherwise it blocks submission or treats the attempt as a failure. In the deployable system, parsing is not required by the server (which verifies $E_{p_i}$); the parse procedures are relevant only to the study prototype and to any client-side UI support.

Repetition trick (exploratory). Although Mindlock included a repetition-based option in the interface, no participants used it in our study. Because repetition can introduce additional structure and potential ambiguity, we treat it as exploratory and move its definition and discussion to Appendix A.

**Server-side storage and check.**

The server *S* stores verifiers $e_i=\textsf{Ver}(E_{p_i})$ and $b_i=\textsf{Ver}(B_{p_i})$.^1^ On login with input *x*,

6 $$\begin{aligned} \textsf{Auth}(\mathrm {ID_i,x})= \left\{ \begin{aligned}&\text {alarm}  &   \text {if } \textsf{Verify}(x, b_i),\\&\text {accept iff } \textsf{Verify}(x,e_i)  &   \text {otherwise.} \end{aligned} \right. \end{aligned}$$

### Per-account data model, alarm policy, and recovery

**Deployment-only note.** The storage and alarm policy in this subsection specify the *intended deployable system* (where the server verifies $E_{p_i}$ and alarms on $B_{p_i}$). These server-side mechanisms were *not* implemented in the Mindlock user study, where websites remained unmodified and verified only the base password $B_{p_i}$.

Per-account data model. Table 1 summarizes what is stored by each party for an account. The design goal is that the password manager (PM) never stores the actual login secret $E_{p_i}$, and the user-held mnemonic $M_i$ is not stored by the server.

**Table 1:** Per-account data model (account *i*)

**Party**	**Stored items (high level)**
Server *S*	Verifier for enhanced password $e_i=\textsf{Ver}(E_{p_i})$; verifier for decoy/base $b_i=\textsf{Ver}(B_{p_i})$; alarm-policy metadata (e.g., cooldown window, last-alarm timestamp, and optional threshold counter).
Password manager (PM)	Base/decoy password $B_{p_i}$ (stored under standard PM protection, e.g., encrypted vault storage).
User	Mnemonic $M_i$ (memorized) and knowledge of the chosen trick (e.g., prefix/suffix).

Alarm policy (deployable system). When a login attempt submits the decoy/base password $B_{p_i}$, the server treats this as a strong signal of PM compromise and triggers an alarm. We keep the policy simple: (i) *alarm on first decoy submission* and (ii) a *cooldown window* to rate-limit repeated alarms and reduce alarm-spam denial-of-service. During the cooldown window, additional decoy submissions are logged but do not trigger repeated notifications.

Notification channel. An alarm can be delivered through any out-of-band channel supported by the service provider (e.g., email/SMS/phone call). The key requirement is that the alert be delivered outside the potentially compromised password manager.

Failure modes and account recovery. False alarms are possible if a legitimate user accidentally submits the base password (e.g., forgets to apply the mnemonic, or the password manager autofill fails and the user proceeds with $B_{p_i}$). In this case, recovery follows standard account reset or re-enrolment: the user completes the service provider’s recovery flow, chooses a fresh base password, and re-establishes the enhanced password by applying a new mnemonic accordingly.

**Study prototype note.** In the Mindlock study, no server-side alarm is triggered because the website never receives $E_{p_i}$; Mindlock strips the mnemonic locally and submits only $B_{p_i}$ for usability evaluation.

### Security Analysis

This subsection discusses security in the *vault-only compromise* setting that motivates our design: an attacker obtains password-manager vault contents (including $B_{p_i}$) but is still constrained by normal online authentication throttling. Under this model, submitting the stolen base password $B_{p_i}$ triggers the decoy alarm, while authenticating successfully requires reconstructing the enhanced password $E_{p_i}$ by guessing the user’s mnemonic (and trick). We emphasize that device/endpoint compromise (e.g., keyloggers, malicious extensions, DOM injection, clipboard monitoring) is out of scope for our claims; if the attacker can observe what the user types during login, they may also learn the mnemonic, as with most password-manager-based schemes.

#### Phishing and URL-hijacking

Password managers (PMs) can reduce exposure to phishing by autofilling credentials only when the visited domain matches the stored entry. Our proposal is compatible with these protections because it builds on the same workflow (PM autofill followed by user action). Attacks that control the endpoint or browser (e.g., malicious extensions, DOM injection, keylogging, screen capture, clipboard monitoring) may capture whatever the user submits at login; these endpoint-compromise threats are explicitly out of scope for our security claims (Section 4.1).

#### Vault-only compromise and online guessing

If an attacker obtains a user’s password-manager vault (e.g., via master-password compromise), they recover the stored base passwords $B_{p_i}$ but not the enhanced login secrets $E_{p_i}$. In the intended deployable system, submitting $B_{p_i}$ triggers an alarm, while successful authentication requires guessing the user’s mnemonic $M_i$ and the selected trick t∈T under the service’s online rate limits. Because user-chosen mnemonics can be predictable, we recommend mitigations such as mnemonic blacklists, a strength meter, and minimum policy requirements to reduce mnemonic guessability and discourage reuse.

Stronger attackers (out of scope). If the attacker can observe the endpoint during login (or obtains a sample of $E_{p_i}$ via phishing or malware), they may learn the mnemonic directly; similarly, if the attacker compromises both the PM and the service provider’s authentication backend, the setting differs from our vault-only model. These stronger attackers are out of scope for the claims we make in this paper.

## Methodology

### User Study Setup

A user study was conducted to evaluate the practicality and effectiveness of our proposed method in enhancing password security. Participants were recruited from multiple academic institutions, with eligibility criteria set at a minimum age of 18. The study was approved by the respective Research Ethics Boards prior to data collection.

**Recruitment and Experimental Design:** The study was advertised to undergraduate and graduate students over 18 from both universities. Participants reviewed and signed a consent form, then received a manual on installing the Mindlock extension and applying mnemonic tricks to their base passwords for various online services (e.g., LinkedIn, Facebook). This research suggests that users familiar with password managers found Mindlock’s mnemonic techniques to be a secure, manageable addition to their routines, allowing for a smooth transition. Over a month-long experiment, 22 participants applied prefixes, suffixes, or repetition to strengthen their passwords. The timeframe allowed users to integrate the techniques into their daily routines. Although the smaller sample limited statistical power, it provided rich qualitative insights, with participants showing clear improvement in mnemonic use over time. At the end of the study, participants submitted Mindlock-collected data and completed exit surveys capturing their experiences and perceived improvements in memorability and ease of use.

### Data Collection and Evaluation Metrics

Our data collection involved both quantitative and qualitative approaches to evaluate how participants interacted with Mindlock.

#### Quantitatively

**Fig. 3:**
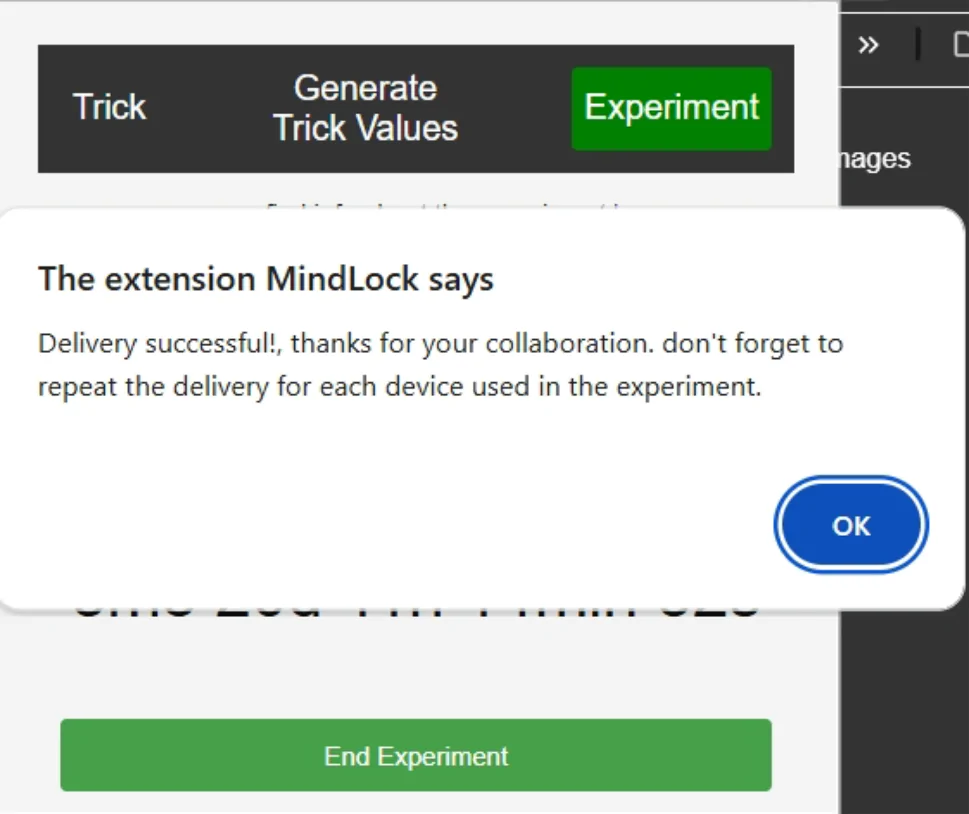
End experiment interface

We captured detailed interactions during the experiment, starting when participants saved their mnemonics and attempted logins. All actions were logged locally until users clicked the “end experiment” button (see Figure  3). At that point, encrypted logs containing the mnemonic type (prefix, suffix, repetition), specific mnemonic, and a website identifier were sent to a secure, research-only server. This allowed us to track memorability and updates, assess login success, and evaluate practical effectiveness under real-world conditions.

#### Qualitatively

After completing the experiment, participants filled out a System Usability Scale (SUS) questionnaire [43] to assess Mindlock’s usability. Users also provided open-ended feedback, offering insight into usability challenges, preferences, and ideas for improvement. This enriched our understanding of the user experience and informed directions for refinement.

#### Evaluation Metrics

- **Success Rate:** Frequency of successful logins using mnemonic-enhanced passwords.
- **Failure Rate:** Frequency of login failures, highlighting mnemonic complexity.
- **Usage Frequency:** Most-used mnemonic trick type (prefix, suffix, repetition), revealing preferences and intuitiveness.
- **Feedback Analysis:** User input on satisfaction, challenges, and feature requests, gathered via questionnaires.
- **Strength Assessment:** Security of mnemonic components evaluated using the Zxcvbn metric [44].

This mixed-method approach enabled us to rigorously assess memorability, usability, and password strength in real-world conditions.

- **Success Rate:** We measure how often participants successfully log into their account using their password enhanced with the mnemonics they have created.
- **Failure Rate:** We measure how often participants failed to log into their accounts. This gives us insights into the complexity of using mnemonics.
- **Usage Frequency:** We track the most popular mnemonic trick type among participants (prefix, suffix, or repetition). Understanding the popularity of each mnemonic type can inform us about participant preference and might suggest which methods are more intuitive for securing passwords.
- **Feedback Analysis:** Through our Questionnaires, we gather feedback about participants’ experience with Mindlock, including their challenges, satisfaction, and recommendations. This feedback gives us an understanding of what they liked and did not like about Mindlock, guiding future improvements and feature additions.
- **Strength Assessment Using Zxcvbn:** We employ the zxcvbn metric [44] to assess the security of the mnemonic components devised by users. Zxcvbn is an advanced password strength estimator that goes beyond simple rule-based metrics. It uses pattern matching and conservative estimation techniques to measure passwords against common tactics found in password-cracking tools and common password lists.

## Usability Results

In our research, we aimed to determine how effective mnemonic techniques are in improving both memorability and security of passwords. Using the Mindlock extension as a substitute for the mnemonic component, we measured various aspects of user interactions, specifically focusing on how participants often employed mnemonics to enhance their passwords successfully, the mnemonic type they preferred, and the overall experience with the system. This section will provide a detailed analysis of how mnemonics, implemented through Mindlock, impacted password management practices among our participants. The results aim to give valuable insights into the practical applications of mnemonic strategies in enhancing cybersecurity measures.

### Success and Failure Rates

The findings from the study directly address **RQ2** in Section 1. The data revealed a success rate of 71.86%, indicating that a significant majority of participants were able to successfully use mnemonic techniques to manage their passwords. This suggests that mnemonics can indeed simplify password management by making passwords easier to remember without necessarily compromising their strength or security. This finding also contributes to answering **RQ1**, as the ease of using mnemonics aligns with **RQ1**’s focus on the trade-off between security and usability, demonstrating that enhanced security can be achieved with minimal impact on usability. Conversely, the observed failure rate of 28.14% highlights critical areas for improvement. This failure rate may be indicative of several potential challenges:

- **User Interface Issues:** There could be some aspects of the Mindlock extension’s user interface that are not clear enough, leading to errors in the mne-monic application.
- **User Habits:** Users may be accustomed to immediately pressing “Enter” after the password manager autofills the password field, inadvertently skipping the mnemonic step. This habitual action may have contributed to initial failures.
- **Complexity of Mnemonics:** Some participants may have chosen complex mnemonics that were difficult to recall after some days had passed, may have required enough time to adapt to integrating mnemonics into their password routines, or possibly lacked sufficient training in effective mnemonic creation and usage.

**Fig. 4:**
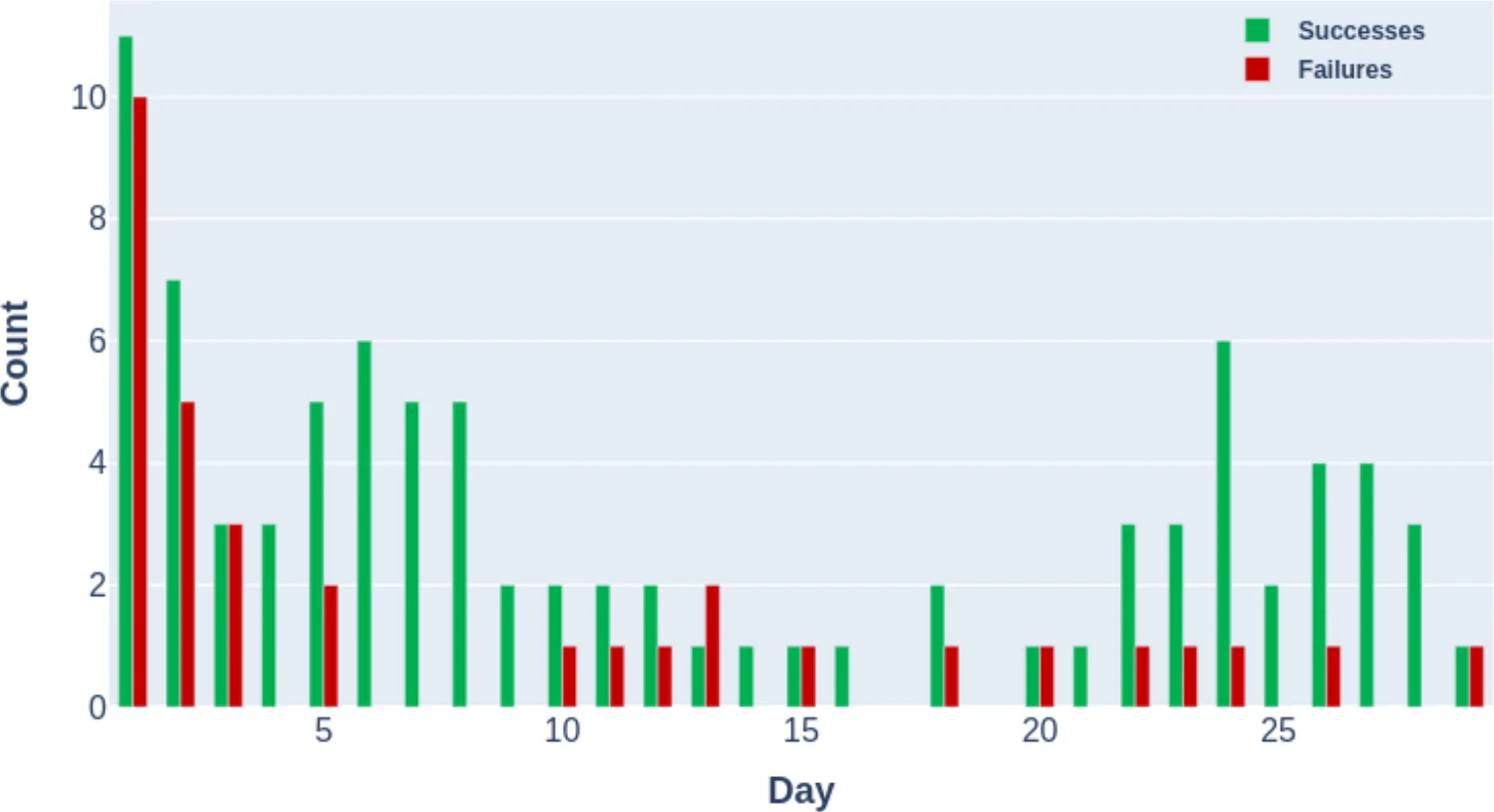
Login results over time

By examining these aspects, the study addresses the question of mnemonic memorability and opens avenues for refining mnemonic strategies to enhance both memorability and security. Notably, as detailed later in Section 6, the failure rate decreases over time (see Figure 4), suggesting that users gradually adapt and overcome initial usability challenges, including habitual missteps.

### Mnemonic Usage Type

This subsection examines which type of mnemonic was mainly utilized by participants, an analysis relevant for understanding user preferences and the practical application of mnemonic techniques in real-world settings. The findings revealed that 73% of participants preferred using prefixes, while 27% chose suffixes. Importantly, participants were free to choose any mnemonic integration strategy, yet none opted for repetition-based patterns (e.g., the “repetition trick”). This absence suggests that users naturally gravitated toward simpler and more intuitive techniques, likely due to their ease of recall and compatibility with existing password habits. While suffixes were less common than prefixes, they were still selected by nearly a third of participants, indicating they remain a viable strategy. These findings underscore the memorability and user-friendliness of straightforward mnemonic approaches, providing additional evidence in addressing **RQ2** in Section 1.

### Statistical Analysis of Mnemonic Type Preferences

A Chi-Square test was performed to further understand the relevance of the distinct preferences for mnemonic types observed among participants. The analysis yielded a Chi-Square statistic of 8.875 and a p-value of 0.003, confirming that the differences in mnemonic type usage are not random but statistically significant.

### Zxcvbn Results

The zxcvbn scores for mnemonics alone are low on average (mean 1.10 out of 4; variance 0.71), indicating that many user-chosen mnemonics are predictable in isolation (Table 2). To make this clearer, we also summarise the distribution over the unique mnemonics we collected (n=44 unique strings): 84.1% fall in the lowest bands (score 0–1), with 20.5% scoring 0 and 63.6% scoring 1. This matters because, under our vault-only threat model, the attacker learns the base password $B_{p_i}$ from the password manager and the main remaining unknown is the mnemonic (and trick). As a result, the security benefit of our approach depends on mnemonic guessability, not on length increase alone. Due to anonymity constraints, we did not collect base passwords or the combined enhanced passwords and therefore do not directly compute zxcvbn on $E_{p_i}$. Instead, we use mnemonic-only zxcvbn as a proxy signal for $G(M_i)$ (Section 4.1) and treat the low scores as motivation for simple mitigations (blacklists, a strength meter, and basic policy rules) that can be enforced locally in the mnemonic interface.

**Table 2:** ZXCVBN score distribution for unique mnemonics (n=44)

Score	Count	Percent (%)
0	9	20.5
1	28	63.6
2	4	9.1
3	2	4.5
4	1	2.3

### Mnemonic predictability: policy and mitigations

Our results show that many mnemonics are weak when evaluated in isolation. Since our security goal is specifically to raise the bar under vault-only compromise (where the attacker already knows $B_{p_i}$), we recommend lightweight mitigations that reduce mnemonic guessability while preserving usability.

Blacklist of common mnemonics. The interface can reject mnemonics that match common patterns. This prevents trivially guessable choices without requiring users to memorize long secrets.

Strength meter and feedback. A local strength meter (e.g., zxcvbn run client-side) can provide immediate feedback and encourage users to adjust their mnemonic. Because scoring is computed locally, this does not require uploading secrets.

Minimum policy (including the ≥5 constraint). We enforce a minimum mnemonic length (at least five characters) as a usability-conscious baseline that filters out extremely short mnemonics while keeping the barrier low enough for adoption. Length alone is not sufficient; therefore, the minimum policy can be paired with simple diversity requirements (e.g., at least two character classes) and/or a minimum zxcvbn score threshold, depending on deployment needs.

**Table 3:** Mnemonic words with ZXCVBN scores and success rates

Mnemonic	Score	Success (%)	Mnemonic	Score	Success (%)
Mnemonic 1	1	80.0	Mnemonic 11	1	100.0
Mnemonic 2	1	33.3	Mnemonic 12	1	66.7
Mnemonic 3	1	100.0	Mnemonic 13	1	57.1
Mnemonic 4	1	83.3	Mnemonic 14	1	42.9
Mnemonic 5	1	100.0	Mnemonic 15	1	88.9
Mnemonic 6	0	75.0	Mnemonic 16	2	100.0
Mnemonic 7	0	50.0	Mnemonic 17	1	100.0
Mnemonic 8	1	50.0	Mnemonic 18	1	66.7
Mnemonic 9	1	62.5	Mnemonic 19	3	57.1
Mnemonic 10	4	100.0	Mnemonic 20	1	62.5

Key takeaway. Security improvements in our threat model depend primarily on mnemonic guessability and reuse patterns, not on added length alone. The mitigations above are intended to increase the effective guessing cost of $M_i$ under online throttling while keeping the password-manager login process simple.

### Per-site vs. global mnemonics

Mindlock allows mnemonics to be set per site or reused across multiple sites, which creates a trade-off between ease of use and reuse risk. A global mnemonic is easier to remember, but if it is learned once, it may help attack other accounts. A per-site mnemonic reduces this risk but increases the number of secrets a user must remember. We recommend a tiered approach: distinct mnemonics for high-value accounts (e.g., email/banking) and limited reuse only for lower-risk accounts. If an attacker learns one mnemonic (e.g., via phishing/endpoint observation), reuse can amplify impact across accounts; per-site (or tiered) mnemonics contain the damage.

### Impact of Mnemonic Complexity on Login Success and Security

Table 3 presents a sample of mnemonic words, their Zxcvbn complexity scores, and login success rates. Simpler mnemonics generally show higher memorability, often nearing 100% success. Notably, “Mnemonic 10” with a high score of 4 also achieved perfect recall, showing that well-designed complex mnemonics can be memorable, especially when tied to personal meaning or familiar patterns.

This highlights an important insight: simple mnemonics, when cleverly combined with other secret elements, can yield strong, user-friendly passwords. This balance is crucial for creating secure, usable password systems.

**Fig. 5:**
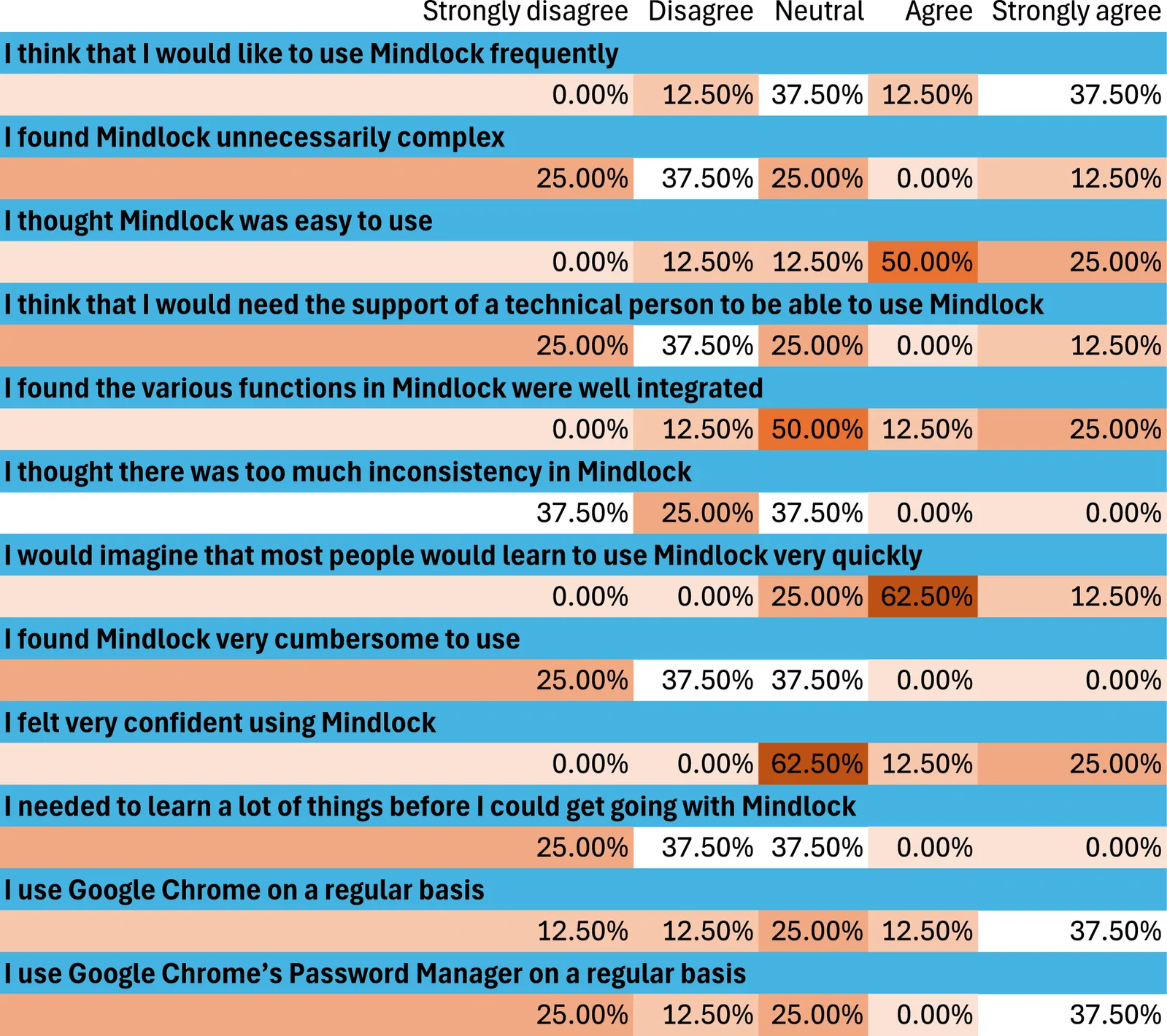
SUS analysis result

### Skill Progression

The evolving performance of users with Mindlock in the study offers a revealing glimpse into how effectively they adopt mnemonic techniques over time, providing a nuanced view of the tool’s usability enhancements. The data shows an intriguing pattern: initially, successes and failures are almost neck and neck, highlighting the learning curve associated with mastering new mnemonic methods. As shown in Figure 4, while the journey begins with a balance between success and failures, there’s a noticeable trend where successes gradually begin to outpace failures. This shift suggests that, over time, users not only get more comfortable with the techniques but also become more adept at integrating them into their routine, leading to a consistent rise in successful outcomes. This pattern underscores the practical utility of Mindlock in fostering stronger password security through user adaptation, suggesting that with sustained use, mnemonic techniques can significantly enhance password management efficiency.

### SUS Results

Figure 5 plots the per–item response distributions for the *SUS questionnaire* (Items 1–10), and Table 4 reports the corresponding item means and overall SUS. The SUS mean is **70.0** (95% CI: **57.2–82.8**; *n*=11 complete responses out of 22 invited; 50% response rate), indicating *acceptable to good* usability. Positively keyed items on ease of use and confidence (Items 3 and 9; means ≈3.9) were strongest, and respondents generally felt they could learn the system quickly (Item 7; 3.73). The negatively keyed items’ complexity, inconsistency, and cumbersomeness (Items 2, 6, 8; means ≈2.0–2.4) were relatively low, suggesting these were not major concerns. The *well–integrated* item (Item 5) was also favourable (mean 3.55). Overall, the SUS profile is positive with some headroom for simplification, and all statistics are based on complete SUS responses.

**Table 4:** System Usability Scale (SUS) results and additional questions. Sample: 22 invited; 11 completed the SUS (50% response). Statistics use complete responses (n=11). 95% CI computed with the *t* distribution ($n\!-\!1=10$)

Item	Statement (short)	Mean (1–5)	SD
1	Use frequently (+)	3.36	1.03
2	Unnecessarily complex (–)	2.36	1.36
3	Easy to use (+)	3.91	1.04
4	Need technical support (–)	2.00	1.27
5	Well integrated (+)	3.55	1.13
6	Too inconsistent (–)	2.09	0.94
7	Learn quickly (+)	3.73	0.65
8	Very cumbersome (–)	2.18	0.98
9	Felt confident (+)	3.91	0.94
10	Need to learn a lot (–)	1.82	0.87
**SUS (0–100)**		**70.0** (95% CI: 57.2–82.8)	
11	Use Chrome (extra)	3.36	1.57
12	Use Chrome PM (extra)	2.73	1.62

(+) positively keyed items; (–) negatively keyed

### Impact on Password Security and Usability

A key objective of this study, as outlined in **RQ1**, was to examine the trade-off between security and usability. User preferences provide evidence for this balance. The 71.86% success rate in mnemonic recall, supported by a significant chi-square result, shows users could apply mnemonics effectively, indicating good usability. From a security perspective, our intended deployable design adds a user-held secret (the mnemonic) on top of the password-manager-stored base password, which can raise the bar in the vault-only, rate-limited setting described in our threat model. Feedback from Figure 5 shows that while some noted an initial learning curve, most found Mindlock easy to incorporate into their routine. Overall, the results suggest the approach can achieve a favorable usability-security trade-off under the scoped threat model.

### Security Analysis and Attack Resistance

Our approach is designed to improve resilience to vault-only password-manager compromise by separating what is stored (the base/decoy password) from what is needed to authenticate (the enhanced password that includes a user-held mnemonic). In the intended deployable system, submitting the base password can trigger an alarm, while successful authentication requires guessing the mnemonic (and trick) under online rate limits. Again, we do not claim protection under endpoint compromise. Within the scoped model, the approach raises the difficulty of unauthorized login attempts while preserving a password-manager-style workflow.

## Discussion

This study suggests that mnemonic tricks can be memorable and usable in practice, achieving a 71.86% success rate. The 28.14% failure rate highlights challenges related to mnemonic complexity and interface design, suggesting the need for further optimization. Participants generally found Mindlock simple and helpful in making password practices more manageable. Suggestions such as offering video tutorials indicate a desire for easier onboarding, while positive feedback highlights its educational impact. Although Mindlock served only as a prototype for testing the proposed mnemonic workflow, user feedback points toward potential improvements for future development. Usability was evaluated through System Usability Scale (SUS) scores and behavioral patterns, revealing user satisfaction and adaptation to mnemonic use over time. However, a detailed end-to-end security evaluation was beyond the study’s scope; our security claims are therefore scoped to the vault-only, rate-limited setting described in our threat model. The strong preference for prefix mnemonics suggests that future designs should prioritize simpler prefix usage while improving support for other mnemonic types. Overall, these findings support the feasibility of mnemonic-based augmentation while maintaining practical usability, rather than proving broad security improvements under stronger attacker models.

### Limitations of the Study

This study is a feasibility pilot with a relatively small sample (22 recruited). Only 11 participants completed the SUS questionnaire (50% response rate), which introduces potential nonresponse bias. Participants were primarily tech-inclined (recruited from academic institutions), so results may not generalize to broader populations. The one-month duration may be insufficient to assess long-term mnemonic retention. Finally, we did not include a control group (e.g., password-manager use without mnemonics), so findings should be interpreted as preliminary evidence of usability feasibility rather than a definitive comparative evaluation.

### Future Research Directions

Future studies should include diverse demographic groups to better understand mnemonic adoption across varying ages, education levels, and technological experiences. Research should also examine users who rely on digital password managers to refine mnemonic techniques for broader applicability.

Further work could simulate a service provider requiring both a base password and a mnemonic during account creation, enabling end-to-end usability testing and security analysis (e.g., under online guessing budgets). Investigating retention beyond one month and exploring the relationship between mnemonic guessability (e.g., zxcvbn) and login success would also strengthen comparisons of success and failure patterns.

## Conclusion

This research presents a password-augmentation approach combining mnemonics with password managers and decoy-based detection to improve resilience to vault-only compromise. By storing only base passwords in the password manager and requiring a user-held mnemonic to form the enhanced secret, the approach aims to mitigate the single point of failure associated with password-manager vault exposure. Mindlock served as a usability prototype, and our month-long study provides preliminary evidence of feasibility and promising usability/adoption signals. Our security claims are scoped to the vault-only, rate-limited setting, and we do not claim protection under endpoint compromise.

## Data Availability

The datasets generated and analyzed during this study contain human subject data and cannot be shared publicly due to privacy/ethics restrictions.

## Repetition trick (exploratory)

Mindlock’s interface included an exploratory repetition option in which a token is inserted at regular intervals. No participants selected this option in our study, and we do not rely on it for our core claims. Because repetition may introduce predictable structure and can create parsing ambiguity (e.g., if the inserted token also appears naturally in the base password), we treat it as exploratory and leave it as future work.

## Footnotes

1 A typical verifier for passwords is a cryptographic hash function. If we call such a function *h*, the verifier for a password *x* is computed as $\textsf{Ver}(x)=h(x)$. The $\textsf{Verify}(x, v)$ boolean function, which checks whether *x* is verified by *v*, is defined as $\textsf{Verify}(x, v):=h(x)=v$.
